# The Universal Standard for Safe and Secure Work with Arthropod Vectors: The American Committee of Medical Entomology’s Arthropod Containment Guidelines

**DOI:** 10.4269/ajtmh.19-0199

**Published:** 2019-04-08

**Authors:** Stephen Higgs

**Affiliations:** Biosecurity Research Institute (BRI), Kansas State University, Manhattan, Kansas

The American Society of Tropical Medicine and Hygiene (ASTMH) is pleased to announce the publication of revised Arthropod Containment Guidelines,^1^ developed by the Society’s subgroup, the American Committee of Medical Entomology (ACME).

Like the original guidelines, these are published in Vector-Borne and Zoonotic Diseases, are open access, and can be viewed and downloaded at https://www.liebertpub.com/doi/10.1089/vbz.2018.2431. An editorial providing some historical perspective is available at https://www.liebertpub.com/doi/10.1089/vbz.2019.29001.hig.^2^

These guidelines have become a universal standard for safe and secure work with arthropod vectors, and are referred to in the highly regarded handbook “Biosafety in Microbiological and Biomedical Laboratories.”^3^ They are the result of tireless effort by dedicated member experts, who painstakingly updated the previous 2003 guidelines, also authored by ACME members. In addition, they solicited comments from many outside subject matter experts. The original guidelines took over 2 years to write and served as the go-to-source for the community. In the 15 years since publication, new technologies and approaches that can be applied to research with arthropods have been developed, and an update was warranted. I have been involved with the guidelines since the idea was first discussed at an ASTMH Annual Meeting. As I am interested in history, with a specific interest in the history of our Society and the fields of arboviruses and medical entomology, this new publication provides an opportunity to briefly describe the very significant contributions of the Society to the field of vector-borne diseases.

The ASTMH has a long history of expertise with pathogens transmitted by arthropod vectors.^4^ William Crawford Gorgas, famous for his pioneering work in Panama to control yellow fever, was the fourth Society President. The Society recently established a new medal named after Clara Southmayd Ludlow,^5^ an entomologist and first woman member of ASTMH. Another medal is named in honor of a famous researcher, Walter Reed, who demonstrated the involvement of mosquitoes in the transmission cycle of yellow fever, and the Harry Hoogstraal medal is awarded for outstanding achievements in medical entomology. Society member Max Theiler was awarded the Nobel Prize in Medicine or Physiology for his work on the development of the vaccine for yellow fever virus that is still in use today.

The *American Journal of Tropical Medicine and Hygiene* celebrated its 100th volume in January this year, having been created in 1952 when the Society’s *American Journal of Tropical Medicine* merged with the Journal of the American Malaria Society. A recent message to our members during Trop History Month highlighted publications from 1952 to 2016 and showed sustained interest in vector-borne diseases, specifically malaria, dengue, leishmaniasis, schistosomiasis, and Chagas disease. Publications on mosquitoes have remained steady for more than a 60-year period, at approximately 5% of all articles published.

In 1961, the Society’s first subgroup, the American Committee on Arthropod-Borne Viruses (ACAV), was formed, and in 1985, the ACME was formed. Working with vector-borne pathogens has always been potentially dangerous, and in 1980, the ACAV Subcommittee on Arbovirus Laboratory Safety published an important document entitled “Laboratory safety for arboviruses and certain other viruses of vertebrates.”^6^ This article included a list of viruses that had been associated with laboratory deaths and importantly provided criteria that could be used to determine the level of practice and containment necessary for safe handling of the viruses. A comprehensive list of arthropod-borne viruses was provided, with risk described on a scale of one to four that has become a basis of virus research to this day. The appendices provided descriptions of recommended competence, practice, and containment levels for arboviruses in laboratories, and importantly for this editorial, discussed procedures for safely working with infected arthropods and some design and operational considerations for insectaries.

Since their formation, both ACAV and ACME continue to be dynamic subgroups within the Society, with dedicated and well-attended symposia at our annual meetings, and other activities throughout the year. Experts in these fields are members of these subgroups, together with many young members from countries around the world. As seen with the recent emergence of chikungunya and Zika viruses, involving hundreds of thousands of cases in multiple countries, we remain unprepared, and often lacking in understanding of pathogens that were described frequently in our journal decades ago. As illustrated above, and with the publication of ACME’s Arthropod Containment Guidelines, the ASTMH continues to lead from the front to bring together global expertise and help in the fight against old and new diseases, wherever and whenever they occur.
